# Editorial: Generating useful genetic variation in crops by induced mutation, volume III

**DOI:** 10.3389/fpls.2023.1301977

**Published:** 2023-10-18

**Authors:** Antonio Costa de Oliveira, Apichart Vanavichit

**Affiliations:** ^1^ Plant Genomics and Breeding Center, Elieu Maciel School of Agronomy, Federal University of Pelotas, Capão do Leão, Brazil; ^2^ Rice Science Center and Rice Gene Discovery, National Center for Genetic Engineering and Biotechnology and Agronomy Department, Faculty of Agriculture, Kasetsart University, Kamphangsaen, Nakhonpathom, Thailand

**Keywords:** induced mutation, TILLING (targeting induced local lesions in genomes) by sequencing, CRISPR, gamma-rays (637), variability

Mutations are the source of variation and variation is the raw material for natural and artificial selection. This Research Topic deals with the useful variations coming from induced mutation techniques. The novel variations add to the existing ones making our progress in developing better and more nutritious plants easier. Overall, our Research Topic has generated material for three volumes, translating its importance to the field of plant genetics and breeding. In this third volume, five papers are presented and gather a range of traits that can be assessed/altered by the use of induced mutations. The paper by Lee et al. addresses elegantly the edition of the Gametophytic Self-Incompatibility system in diploid potato, in order to fix favorable alleles and heterotic potential. The S-RNase and HT genes shown previously to contribute to GSI in the Solanaceae family, were target of a CRISPR-Cas9 to knockout HT-B either individually or in concert with S-RNase in the diploid self-incompatible *S. tuberosum* clone DRH-195. Different levels of seed numbers were obtained with the different knockouts indicating a synergistic effect between HT-B and S-RNase in self-compatibility in diploid potato. The subject is a hot topic among potato breeders. Self-compatibility, if well worked out, could uncover an enormous reservoir of genetic variability, making a whole new level of opportunities for potato breeding. Wang et al. identified a new dwarf gene in wheat (jg0030). This mutation was induced by gamma-ray mutagenesis of the wheat variety ‘Jing411’ (wildtype). Compared with ‘Jing411’, plant height in the jg0030 mutant was reduced by 7%-18% in two years’ field experiments, and the plants showed no changes in yield-related traits. Plant height is one of the most important agronomic traits that affects yield in wheat, owing to that the utilization of dwarf or semi-dwarf genes is closely associated with lodging resistance. The mutant jg0030 is a GA-sensitive mutant controlled by a major gene mapped on the long arm of chromosome 2B. The authors bring an interesting trait for crop production. The climate changes ahead of us will test our ability to have more resilient and robust genotypes to cope with adverse effects resulting from these environmental changes. Novel short stature plants can be of great help in these scenarios. Mubarak et al. selected for mutations regarding low photorespiration. According to the authors, the mean chlorophyll loss in WTs ranged from 65% to 11%, respectively, whereas in the mutant lines, chlorophyll losses ranged from 0 to 100%, suggesting considerable phenotypic variation. Rice mutants with a reduced chlorophyll reduction (<10%) were identified as ‘Chlorophyll retention mutants’(CRMs) under low CO2 stress. The potential of these mutants relates to breeding rice with improved primary metabolism. One of the critical changes due to Global Warming will be hotter nights, where we expect plants to perform respiration and burn glucose, generating Co2. These chlorophyll retention mutants may help us to better understand these mechanisms and modulate photorespiration. Szurman-Zubrzycka et al. bring attention to an effective tool for mutation breeders, TILLING (Targeting induced local lesions in genomes). The comparison of TILLING and CRISPR/Cas, through its advantages and disadvantages, make us feel that we still need TILLING as a support tool in finding useful mutations that can be translated into useful genotypes. The occurrence of background mutations is pointed as a downside. However, the technique has evolved with the help of other technologies, such as NGS. As explained by the authors, a technique that can (with some change in detection sensitivity) be performed in a simple lab, with very simple equipment or with improved performance using modern and more sophisticated equipment. The authors take the reader to many good examples where this technique was successful. Zhai et al. describe the functional analysis of polyphenol oxidase 1 gene in common wheat. Polyphenol oxidase (PPO) is a major cause of the undesirable brown color of wheat-based products. The authors provide the function and genetic regulation of Ppo1 using TILLING and RNAi (RNA interference) techniques. The authors describe reductions in Ppo1 transcripts (15.5-60.9%) to result in reductions of PPO activity (12.9-20.4%). Two highly conserved sites, where the frequency of glycine was 94.6% and 100%, respectively, adjacent to the entrance of the hydrophobic pocket of the active site, when mutated, generated significant phenotypes. Mutants M092141 and M091098 are claimed to be important germplasm sources for improving PPO activity. Overall, the manuscripts deal with harnessing useful variation in order to develop improved crops resilient to climate changes and their unfolding consequences.

## Author contributions

Ad: Writing – original draft, Writing – review & editing. AV: Writing – review & editing.

